# High Sensitivity of Circulating Tumor Cells Derived from a Colorectal Cancer Patient for Dual Inhibition with AKT and mTOR Inhibitors

**DOI:** 10.3390/cells9092129

**Published:** 2020-09-20

**Authors:** Daniel J. Smit, Laure Cayrefourcq, Marie-Therese Haider, Nico Hinz, Klaus Pantel, Catherine Alix-Panabières, Manfred Jücker

**Affiliations:** 1 Institute of Biochemistry and Signal Transduction, University Medical Center Hamburg-Eppendorf, Martinistraße 52, 20246 Hamburg, Germany; d.smit@uke.de (D.J.S.); hinz_nico@gmx.de (N.H.); 2 Laboratory of Rare Human Circulating Cells (LCCRH), University Medical Center of Montpellier, 34093 Montpellier, France; l-cayrefourcq@chu-montpellier.fr (L.C.); c-panabieres@chu-montpellier.fr (C.A.-P.); 3 Molecular Skeletal Biology Laboratory, Department of Trauma, Hand and Reconstructive Surgery, University Medical Center Hamburg-Eppendorf, 20246 Hamburg, Germany; m.haider@uke.de; 4 Department of Tumor Biology, University Medical Center Hamburg-Eppendorf, Martinistraße 52, 20246 Hamburg, Germany; pantel@uke.de

**Keywords:** circulating tumor cells, CTCs, drug sensitivity assays, PI3K/AKT/mTOR pathway, AKT, MK2206, RAD001, dual targeting, colorectal carcinoma, targeted therapy, personalized medicine

## Abstract

Circulating tumor cells (CTCs) are cells shed from the primary tumor into the bloodstream. While many studies on solid tumor cells exist, data on CTCs are scarce. The mortality of cancer is mostly associated with metastasis and recent research identified CTCs as initiators of metastasis. The PI3K/AKT/mTOR signaling pathway is an intracellular pathway that regulates essential functions including protein biosynthesis, cell growth, cell cycle control, survival and migration. Importantly, activating oncogenic mutations and amplifications in this pathway are frequently observed in a wide variety of cancer entities, underlining the significance of this signaling pathway. In this study, we analyzed the functional role of the PI3K/AKT/mTOR signaling pathway in the CTC-MCC-41 line, derived from a patient with metastatic colorectal cancer. One striking finding in our study was the strong sensitivity of this CTC line against AKT inhibition using MK2206 and mTOR inhibition using RAD001 within the nanomolar range. This suggests that therapies targeting AKT and mTOR could have been beneficial for the patient from which the CTC line was isolated. Additionally, a dual targeting approach of AKT/mTOR inside the PI3K/AKT/mTOR signaling pathway in the colorectal CTCs showed synergistic effects in vitro. Depending on the phenotypical behavior of CTC-MCC-41 in cell culture (adherent vs. suspension), we identified altered phosphorylation levels inside the PI3K/AKT/mTOR pathway. We observed a downregulation of the PI3K/AKT/mTOR signaling pathway, but not of the RAS/RAF/MAPK pathway, in CTCs growing in suspension in comparison to adherent CTCs. Our results highlight distinct functions of AKT isoforms in CTC-MCC-41 cells with respect to cell proliferation. Knockdown of AKT1 and AKT2 leads to significantly impaired proliferation of CTC-MCC-41 cells in vitro. Therefore, our data demonstrate that the PI3K/AKT/mTOR signaling pathway plays a key role in the proliferation of CTC-MCC-41.

## 1. Introduction

Most deaths in cancer are associated with metastasis of cells from the primary tumor [1]. During the metastatic process malignant cancer cells have the ability to access the lymphatic system, blood vessels or surrounding tissue [2]. Although the importance of cancer cell metastasis has been shown in the past [3], the entire process is not completely understood yet.

Recently, circulating tumor cells (CTCs), which are shed from the primary tumor into the blood were successfully isolated and taken into cell culture, promising a valuable and robust model to study cancer cell metastasis [4,5]. The CTC cell culture allows insights into the cells responsible for metastasis and opens up a new field in the cancer research area. However, CTC isolation is still challenging due to the low number of cells in the patients’ bloodstream [6,7]. Nevertheless, the possible clinical applications of CTCs are broad and include therapy monitoring [8,9,10], studies on resistance mechanisms [11,12,13], risk stratification for relapse [14,15,16] and potential targets for personalized medicine [17,18]. In some cancer entities, including pancreatic cancer, breast cancer and colorectal cancer, CTC counts are linked to an unfavorable prognosis [14,19,20], highlighting the impact of CTCs in disease progression.

Colorectal cancer (CRC) accounted for over one million cases and half a million death in 2018, making the disease the third most commonly diagnosed cancer entity worldwide [21]. Despite the extensive effort for early detection of CRC in developed countries [21], one out of five patients suffers from metastatic CRC at first diagnosis [22]. In colon cancer metastases can be frequently detected in different organs including the liver, lung, bone and brain. Treatment for metastatic colon cancer remains palliative and only around 14% of these patients survive the disease for more than five years after diagnosis [23].

In the past, the correlation of clinicopathological parameters and presence of CTCs could be established [24]. Furthermore, the presence of CTCs was linked to poor survival in a meta-analysis and CTCs were described as an independent prognosticator in CRC [25]. However, further characterization of CTCs is still limited by the low number of CTCs [26]. The recent establishment of cell lines from CTCs of cancer patients has therefore opened a new avenue for functional analyses of CTCs [4,27,28]. The CTC-MCC-41 colon CTC line that we analyzed in this study was the first long-term stable CTC line established from a patient with colon cancer. The CTC-MCC-41 line was derived from the blood sample of a 57-year-old male patient after first diagnosis prior to treatment of the metastatic colon cancer. In addition to various cancer infiltrated abdominal and mediastinal lymph nodes, the patient suffered from a solitary synchronous liver metastasis at first presentation. 

The cell line features an intermediate epithelial-mesenchymal phenotype with stem cell-like characteristics and the ability to induce endothelial cell tube formation in vitro [4]. Genomic analysis of CTC-MCC-41 cells revealed that *PIK3CA*, *AKT*, *PTEN*, *KRAS* and *NRAS* were wild type, but the cell line harbors a *BRAF*^V600E^ mutation with a stable allele frequency of 50% [29]. The *BRAF*^V600E^ mutation detected in CTC-MCC-41 cells matches the mutational status of the primary tumor and the lymph node metastasis [4,29]. Notably, CTC-MCC-41 has the capacity to form xenograft tumors in mice after subcutaneous injection [4] with matching mutational profile underlining the tumorigenic potential of CTCs as reported in the past [30]. Moreover, in-depth transcriptional analysis has revealed that this CTC line has a distinct transcriptomic signature, not observed in established CRC lines (e.g., HT29) of other sources [4,31], suggesting that CTCs have unique properties that enable them to survive in the bloodstream.

The PI3K/AKT/mTOR signaling pathway not only plays a crucial role in physiologically cell biology, but also in cancer as it regulates cell proliferation, survival, migration, angiogenesis and the utilization of nutrition [32]. Disruption of the tightly regulated PI3K/AKT/mTOR signaling induced by genetic alternations in cancer leads to growth advantages and common cancer-associated patterns [33] such as accelerated cell growth without negative feedback, increased survival and metastatic potential [34]. Therefore, the PI3K/AKT/mTOR signaling pathway became a popular target for new drugs in cancer [35,36,37]. For full activation of AKT, phosphorylation at serine residue 473 is required [38]. Upon full activation, AKT further regulates various downstream substrates inside the PI3K/AKT/mTOR pathway [38,39]. In CRC, a phosphorylation at this site could be detected in nearly half of the CRC samples and was correlated with a poor disease outcome [40].

In the present study, we assessed the functional role of the PI3K/AKT/mTOR signaling pathway in the permanent CTC line CTC-MCC-41 [4]. Further understanding of the tumor cells that are said to initiate metastasis and their signaling is of high importance in order to develop new treatment strategies. Here, we report that the PI3K/AKT/mTOR signaling pathway, which is frequently hyperactivated in cancer, also exhibits an important role in CTCs and may be a valuable target for anti-cancer therapies. We demonstrate strong synergistic effects on the growth of CTCs in vitro by dual targeting AKT and mTOR with MK2206 [41] and RAD001 [42] within the nanomolar range compared to single agent treatment, suggesting a novel therapeutic strategy. Moreover, we could identify differential functions of AKT isoforms on the growth of CTC-MCC-41 line after shRNA mediated knockdown, indicating a crucial role of AKT in this subset of more aggressive tumor cells.

## 2. Results

### 2.1. Strong Activation of the PI3K/AKT/mTOR Signaling Pathway in CTC-MCC-41 

**Figure 1:**
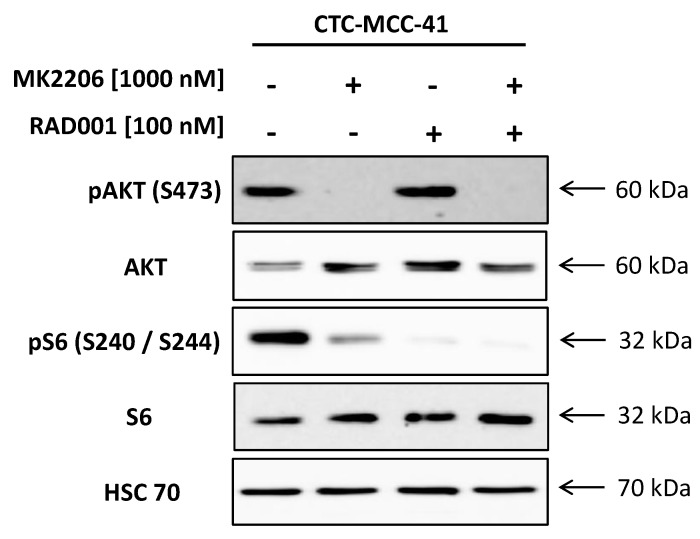
Activity of the PI3K/AKT/mTOR signaling pathway in CTC-MCC-41 and the inhibition of AKT/mTOR axis. The whole cell population of CTC-MCC-41 line (i.e., adherent and suspension cells) was treated for 24 h with AKT inhibitor MK2206 [1000 nM], mTOR inhibitor RAD001 [100 nM], the combination of both [MK2206 1000 nM/RAD001 100 nM] or solvent DMSO. Primary specific antibodies against pAKT (S473), pS6 (S240/S244), pan AKT and S6 were used to access the activity of the pathway and confirm the inhibition of the respective target. Housekeeping protein HSC70 was used as a loading control.

We examined the activation of the PI3K/AKT/mTOR signaling pathway by western blotting (Figure 1). The CTC-MCC-41 line shows a strong phosphorylation of AKT (S473) and of the mTOR downstream substrate S6 (S240/S244). In the next step, we treated the cells with the allosteric pan-AKT inhibitor MK2206, the mTOR inhibitor RAD001 or a combination of both drugs in vitro. The CTC line showed an impaired activation of AKT (S473) and mTOR downstream substrate S6 (S240/S244) after treatment, indicating a potential treatment target. Inhibition of AKT alone by MK2206 completely suppressed phosphorylation of AKT (S473) and diminishes further downstream signaling as measured by pS6 (S240/S244) in CTC-MCC-41 cells. A dual targeting approach of AKT/mTOR was able to further decrease pS6 (S240/S244) level in the CTCs compared to single treatment with MK2206. The treatment with RAD001, either as a single treatment or as a dual targeting approach by additionally targeting upstream AKT, resulted in a strong decrease of pS6 (S240/S244) levels. However, neither the single admission of RAD001 nor the combination therapy was able to completely suppress pS6 (S240/S244) signaling in CTC-MCC-41 line. When targeting mTOR by using RAD001, we did not detect an increase in phosphorylated AKT (S473) as propagated in the past due to feedback loops. 

**Figure 2:**
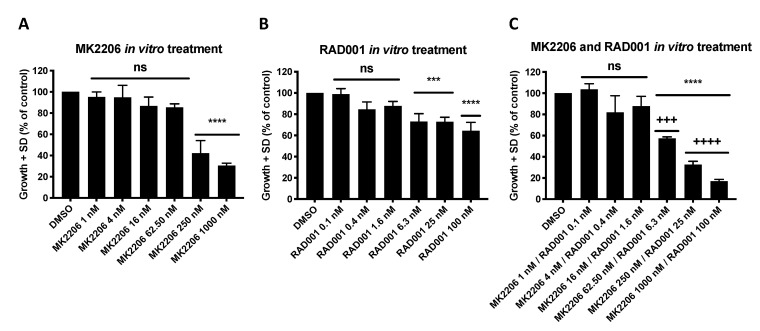
Inhibition of the PI3K/AKT/mTOR signaling pathway inhibits tumor cell growth of CTC-MCC-41 in vitro. The whole cell population of CTC-MCC-41 line (i.e., adherent and suspension cells) was seeded in triplicates in a density of 3000 cells per well into a 96 well flat bottom cell culture plate. The next day MK2206 (**A**), RAD001 (**B**) or the combination of both inhibitors (**C**) was added to the cells and the plate transferred into the IncuCyte^®^ Zoom Live Cell Analysis System. Cell confluence was measured every two hours. Analysis of data was performed based on cell confluence using the provided manufacturers IncuCyte^®^ Zoom Software. The data points were extracted from one representative time point during the exponential growth phase (*t* = 110 h). *p* values were calculated using one-way ANOVA with Dunnett’s multiple comparisons test (ns *p* > 0.05; *** *p* ≤ 0.001; **** *p* ≤ 0.0001). Combination indices (CI) were calculated according to the Chou and Talalay method (++++ strong synergism CI 0.1–0.3; +++ synergism CI 0.3–0.7). The mean values (*n* = 3) with standard deviation are shown.

Next, the effect of MK2206 and RAD001 on proliferation of the colon CTC line was measured under varying concentrations of allosteric AKT inhibitor MK2206 and mTOR inhibitor RAD001 ranging from 1 nM to 1000 nM (MK2206) and from 0.1 nM to 100 nM (RAD001), respectively (Figure 2). A dose-dependent inhibition of CTC growth was observed in comparison to untreated cells with IC_50_ values within the nanomolar range. The results indicate a high sensitivity of the colorectal CTC line CTC-MCC-41 for inhibition of the PI3K/AKT/mTOR signaling pathway by MK2206 and RAD001. 

Single targeting of either AKT or mTOR by MK2206 (IC_50_: 186 nM) or RAD001 (IC_50_: 2.6 nM) in CTC-MCC-41 (Figure 2A,B) showed a high sensitivity for the inhibitor. However, dual targeting of the AKT/mTOR axis was superior to single inhibition and could further inhibit the colon CTC line growth in the combinatory treatment. The analysis of combination indices, according to the Chou and Talalay method [43], revealed synergistic (+++) to strong synergistic (++++) effects in CTC-MCC-41 cells in concentrations ranging from 62.50 nM/6.3 nM (MK2206/RAD001) to 1000 nM/100 nM (MK2206/RAD001) (*p* ≤ 0.0001) (Figure 2C). 

### 2.2. Differential PI3K/AKT/mTOR Signaling in Suspension and Adherent Phenotype of CTC-MCC-41 Cells

**Figure 3:**
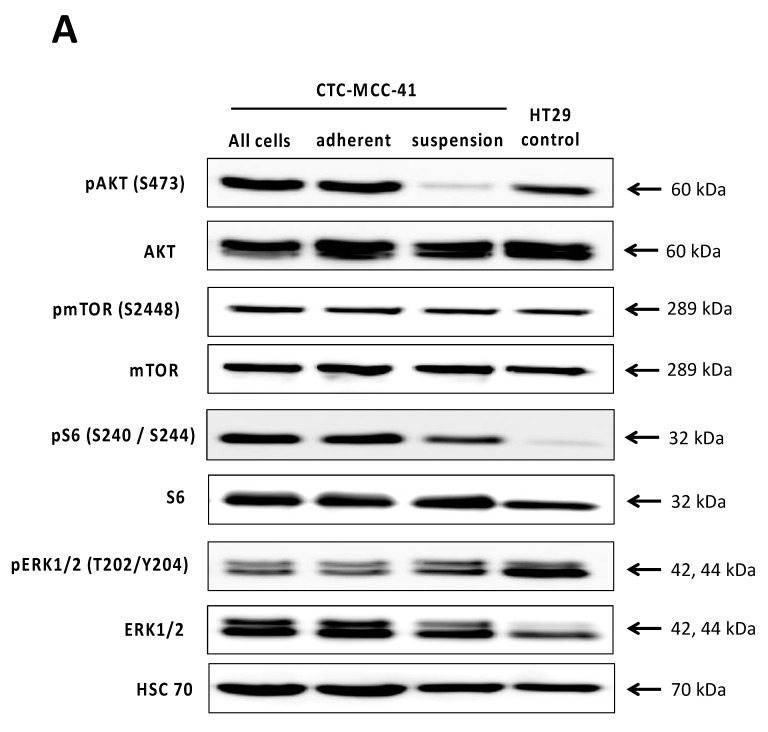

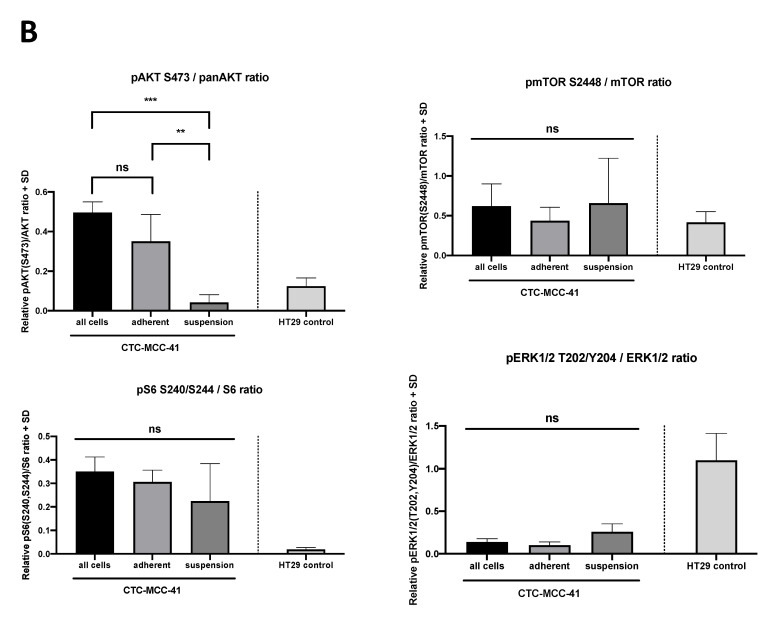
Differential activity of the PI3K/AKT/mTOR signaling pathway in suspension and adherent phenotype of CTC-MCC-41. (**A**) CTC-MCC-41 adherent and suspension cells were separated in the cell culture and subjected to western blot analysis. Whole cell lysates (also referred to as whole population) of CTC-MCC-41 and colorectal cancer cell line HT29 cells were used as control. Primary antibodies against mTOR, pmTOR (S2448), AKT, pAKT (S473), ERK1/2, pERK1/2 (T202/Y204), S6 and pS6 (S240/244) were used to analyze the activity of the RAS/RAF/MEK/ERK and the PI3K/AKT/mTOR signaling pathway. HSC70 was used as a loading control for equal protein loading. (**B**) Densitometric quantification of the western blot analysis as shown in (**A**). The mean values of relative protein activity (*n* = 3) normalized to HSC70 loading control with SD are shown. *p* values were calculated using one-way ANOVA with Tukey’s multiple comparisons test (ns *p* > 0.05; ** *p* ≤ 0.01; *** *p* ≤ 0.001).

To further investigate the activity of the PI3K/AKT/mTOR signaling pathway and other pathways that frequently interact with PI3K/AKT/mTOR signaling, such as the RAS/RAF/MEK/ERK signaling pathway, we conducted further western blot analysis on the CTC-MCC-41 cells (Figure 3). As the cells show a biphasic phenotype in cell culture (suspension vs. adherent), we separated the suspension and adherent fraction particularly. Comparing the whole population, the adherent and the suspension cell fraction, we detected differences restricted to the pAKT (S473) levels (Figure 3A). While the adherent cells show a strong activation of AKT (S473) and therefore matching the whole cell population, the suspension fraction shows significantly decreased pAKT (S473) levels compared to all cells (*p* = 0.0005) and the adherent fraction (*p* = 0.0055) (Figure 3B). No significant differences could be observed in pmTOR (S2448), pERK1/2 (T202/Y204) and pS6 (S240/S244) with respect to the fractions and the whole population. However, we found that CTC-MCC-41 in general showed a strong activity of mTOR, AKT, ERK1/2 and S6. Comparing the whole cell lysate to another solid colorectal cancer cell line, namely HT29 cells, we detected significant higher levels of pAKT (S473) (*p* = 0.0017) and pS6 (S240/S244) (*p* = 0.0082), but not of pmTOR (S2448) (*p* = 0.8729) in the CTCs. Interestingly, pERK1/2 (T202/Y204) expression was significantly higher (*p* = 0.0005) in HT29 control and lower among the whole population, as well as the suspension and adherent fraction of CTC-MCC-41. 

### 2.3. AKT Isoform Specific Signaling in CTC-MCC-41 Cells

**Figure 4:**
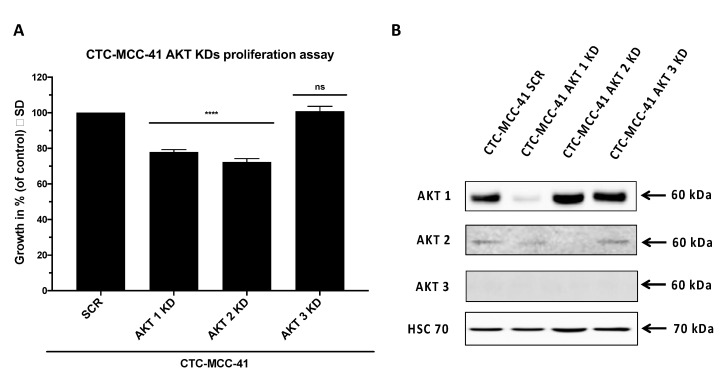
AKT1 and AKT2 knockdown significantly decreases CTC growth in vitro. (**A**) AKT isoform specific shRNA mediated knockdowns (KD) of the whole cell population (i.e., adherent and suspension cells) of CTC-MCC-41 and scrambled (SCR) control were seeded in triplicates in a density of 3000 cells per well into a 96 well flat bottom cell culture plate and placed into the IncuCyte^®^ Zoom Live Cell Analysis System. Cell confluence was measured every two hours. Analysis of data was performed based on cell confluence using the provided manufacturers IncuCyte^®^ Zoom Software. The data points were extracted from one representative time point during the exponential growth phase (*t* = 90 h). *p* values were calculated using one-way ANOVA with Dunnett’s multiple comparisons test (ns *p* > 0.05; **** *p* ≤ 0.0001). The mean values (*n* = 3) with standard deviation are shown. (**B**) Confirmation of stable AKT isoform specific shRNA mediated knockdown of AKT1, AKT2 and AKT3 in CTC-MCC-41 using western blot analysis. A scrambled (SCR) vector served as control. AKT3 signal could not be detected in CTC-MCC-41 cells.

To further verify the hypothesis of a distinct role of AKT isoforms in the CTC-MCC-41 line we generated stable lentiviral isoform specific AKT knockdowns of AKT1, AKT2 and AKT3 in CTC-MCC-41 cells. In proliferation assays we could detect an impaired growth of CTC-MCC-41 cells harboring AKT1 (*p* < 0.0001) and AKT2 knockdown (*p* < 0.0001) compared to scrambled control (Figure 4A). The efficacy of the AKT knockdown was 89% and 91% for AKT1 and AKT2, respectively (Figure 4B). We were not able to detect AKT3 expression in CTC-MCC-41 cells by western blotting. Matching the findings from the western blot analysis (Figure 4B), no significant difference on the proliferation in cells, stably expressing the AKT3 knockdown vector (*p* = 0.9754) compared to control was observed (Figure 4A). To analyze the possibility that growth inhibition of CTC-MCC-41 cells after AKT knockdown was due to increased apoptosis, we conducted FACS analysis and PI/Annexin V FITC staining (Supplementary Figure S1). The apoptosis rates did not significantly differ between the AKT isoform specific knockdowns. Additionally, we could detect an equal viability among the cells. Therefore, we conclude that growth inhibition of CTC-MCC-41 cells was not due to increased apoptosis after AKT isoform specific knockdown.

## 3. Discussion

In this work, we provide first evidence on a crucial role of PI3K/AKT/mTOR signaling in CTC-MCC-41 cells. The sensitivity towards the allosteric AKT inhibitor MK2206 and the synergistic effects on the proliferation by additional targeting mTOR using RAD001 in the colon CTC line provides a rationale for dual targeting the AKT and mTOR axis as postulated earlier [44,45,46,47,48]. The complexity of signaling pathway networks including the PI3K/AKT/mTOR and the RAS/RAF/MEK/ERK pathway makes it difficult to choose the right treatment without triggering crosstalk [49]. Aberrant activation of the PI3K/AKT/mTOR pathway in CRC has been associated with carcinogenesis, early metastatic formation as well as maintenance of established CRC [50]. High levels of phosphorylated AKT (S473) have been reported in 46% of CRC samples and could be linked to increased proliferation, reduced apoptosis and poor clinicopathological outcome including lymph node metastasis and venous vessel invasion [40]. Additionally, a study by Johnson et al., revealed a significantly upregulation of Akt1, Akt2, pmTOR (S2448) and pS6 (T389) in colorectal cancer samples compared to normal tissue of the same patient [50]. 

Accounting for the high prevalence of *PIK3CA* mutations in one third of all CRCs [51,52], combined targeting of AKT and downstream substrate mTOR will be necessary to maintain the inhibition of the signaling pathway [53]. 

Interestingly, mTOR is not only a downstream substrate of AKT, but also obligatory for full activation of the AKT activity by catalyzing the phosphorylation at serine residue 473 [32]. Dual targeting of both AKT and mTOR therefore provides a stronger inhibition of the PI3K/AKT/mTOR signaling pathway and fewer possible escape mechanisms than single drug therapy. The efficacy of RAD001 was previously examined in a phase II clinical trial (NCT00419159) as a single agent therapy for CRC patients who relapsed on conventional treatment regimens. Although the high frequency of PI3K/AKT/mTOR pathway mutations in CRC and the results from a phase I study promised a robust anti-tumor activity, RAD001 failed to prevent disease progression in a phase II clinical trial [54]. The authors emphasized that a combination therapy may be beneficial as mTOR inhibition alone may trigger negative feedback loops inside the pathway leading to increased upstream signaling [49,54]. Therefore, the efficacy of combinatorial inhibition of CTCs in vitro using MK2206 and RAD001 should be further examined and complemented by in vivo data in xenograft models. 

Drug sensitivity screening using CTCs has been conducted in the past [27,28,55]. In 2014, Yu et al. were the first to establish drug sensitivity testing on six stable CTC lines in vitro as well as in a xenograft mouse model in vivo [27]. Pearl et al. were able to successfully predict the sensitivity and response to treatment using CTCs derived from patients suffering from ovarian cancer [55]. Moreover, the authors could successfully confirm the prediction for 13 out of 13 patients in a retrospective analysis [55]. Recently, Koch et al. established a permanent CTC line, namely CTC-ITB-01, derived from a metastatic breast cancer patient. CTC-ITB-01 line exhibited tumorigenic and metastatic capacity in a xenograft mouse model in vivo and remained estrogen receptor positive in culture. Interestingly, the CTC-ITB-01 conserved the same characteristics as the CTCs in the patient. In an in vitro drug sensitivity assay the authors detected a high sensitivity of CTC-ITB-01 for CDK4/6 inhibition using Palbociclib. The high sensitivity of Palbociclib, which is now routinely used in patients who relapsed on prior endocrine therapy, may implicate that the patient the CTC were derived from may could have benefitted from treatment with Palbociclib [28]. Hence, these studies and the model established by Koch et al. indicate the potential of preclinical drug screening with CTCs which should be extensively performed and validated to be implemented into the clinics. 

In addition to the drug sensitivity assays concerning the PI3K/AKT/mTOR pathway, we successfully established stable lentiviral knockdowns of AKT1, AKT2 and AKT3 to investigate the functional role of these AKT isoforms in the CTC-MCC-41 line. In the past years, the distinct functions of AKT isoforms were part of extensive research [56,57,58]. In agreement with previously published findings on the effect of AKT1 on the proliferation of solid tumor cells [56,59], as well as on disseminated tumor cells [60], we observed a decrease in cell proliferation as a consequence of AKT1 knockdown. Although mutations in AKT are rare events in primary CRC samples [61], an oncogenic effect of the AKT1 missense mutation E17K leading to increased signaling through constitutive activation has been reported in CRC [62]. A study on breast cancer CTCs conducted by Aktas et al. was recently able to identify AKT2 expression as a marker of epithelial-mesenchymal-transition (EMT) in more than 60% of CTC positive samples analyzed [63]. The functional role of AKT2 in the physiologically state and in cancer cells has been reported as opposing compared to AKT1 [64,65]. While AKT1 activation directly stimulates cell growth by phosphorylation of p21 and therefore disinhibiting G1-S-transition, AKT2 activation promotes cell cycle exit through p21 binding, therefore competing with AKT1 phosphorylation [64]. However, in our experiments, we observed a significant reduction of CTC-MCC-41 proliferation in vitro after AKT2 specific knockdown, suggesting a crucial role of the kinase in these metastases-competent CTCs. In line with our data, other groups reported increased cell death and suppressed proliferation in CRC cell lines [66,67] and further entities of cancer after AKT2 specific knockdown [68,69]. As Grottke et al. reported, knockdown of AKT3 promoted migration and metastasis in breast cancer cells [70]. Therefore, a reduced or even undetectable expression of AKT3 may be interpreted as a result of downregulation in metastasizing CTCs. 

The CTC-MCC-41 line has been described to express ALDH1 and CD133, two common markers of stem cells [4,29,71]. Additionally, expression of EMT inducer SNAIL1 indicates an intermediate epithelial/mesenchymal phenotype which is in line with the phenotype of the cell line in culture reversing from adherent to suspension cells and vice versa [4]. Moreover, separate cultivation of one fraction, either the suspension or adherent CTCs alone, gives rise to the respective counterpart. One possible explanation in the context of CTCs for this morphology in vitro may be that it reflects the situation in the bloodstream of metastatic patients. The suspension phenotype may reflect those cells who have gone through EMT, while the adherent cells potentially mimic the cells after mesenchymal–epithelial transition. In our experiments we observed diminished phosphorylated AKT (S473) levels exclusively in the suspension fraction of CTC-MCC-41 compared to the adherent fraction and whole cell population. Our data are in line with results from Salt et al. [72] who induced EMT in non-small cell lung cancer cells and also could detect lower pAKT levels after EMT. This may support the hypothesis of a crucial role of EMT in CTCs that has been frequently discussed in the past [73]. Additionally, the lower levels of phosphorylated AKT (S473) in the suspension fraction may implicate a dormant state, as this fraction seems to mimic the properties of CTC in the patient’s bloodstream. The idea of a dormant state with only basic metabolism rate without proliferation has already been proposed in the past [74]. However, as only one permanent CTC line was used in this study, the crucial role of the AKT/mTOR axis in colorectal cancer CTCs should be confirmed in the future when more long-term stable CRC CTC lines are available. 

In conclusion, we functionally assessed the PI3K/AKT/mTOR pathway in a permanent CTC line, namely CTC-MCC-41, derived from a patient with colorectal cancer. In our AKT isoform specific knockdown we demonstrated that AKT1 and AKT2 play a crucial role with respect to proliferation in this colon CTC line. Additionally, we could identify the inhibition of AKT and mTOR in CTCs as a novel druggable target in colorectal cancer. Especially the dual inhibition of AKT and mTOR led to strong decrease of CTC-MCC-41 cell growth in vitro. Although further validation of drug sensitivity screening and comparison with the primary tumor site are necessary before implementation into the clinical routine, the inhibition of CTCs may be a valuable future target to block metastatic progression in CRC patients. 

## 4. Materials and Methods 

### 4.1. Standard Cell Culture

The colon CTC-MCC-41 line was established in the laboratory of rare human circulating cells at the University Medical Center of Montpellier, France [4] and transferred to University Medical Center Hamburg-Eppendorf, Germany. For CTC cultivation, Roswell Park Memorial Institute (RPMI)-1640 media (#11875-093, Thermo Fisher Scientific Inc., Waltham, MA, USA) supplied with 10% fetal calf serum (FCS) (#26140-079, Thermo Fisher Scientific Inc., Waltham, MA, USA) 1% penicillin/streptomycin mix (#15140-122, Thermo Fisher Scientific Inc., Waltham, MA, USA) 1% L-glutamine (#25030-081, Thermo Fisher Scientific Inc., Waltham, MA, USA), 1% insulin-transferrin-selenium-A supplement (100×) liquid (#51300-044, Thermo Fisher Scientific Inc., Waltham, MA, USA), 10 ng/mL human FGF2 (#130-093-839, Miltenyi Biotech, Bergisch Gladbach, Germany), 50 ng/mL human EGF (#130-097-749, Miltenyi Biotech, Bergisch Gladbach, Germany), 0.1 µg/mL hydrocortisone (#74142, Stemcell Technologies Inc., Vancouver, Canada) was used. CTCs were routinely cultivated in non-adherent cell culture flasks (Greiner Bio-One, Kremsmünster, Austria) and passaged by separating the cells growing adherent from the cells growing in suspension. The supernatant was transferred into a falcon tube and centrifuged at 650 g for 5 min at room temperature (RT). In the meanwhile, the cells adherent to the flask were washed with 5 mL PBS and then incubated with Accutase^®^ solution (#A6964, Sigma Aldrich, St. Louis, MO, USA) for 5 min at 37 °C, 5% CO_2_ in the incubator. Suspension fraction cells were washed with PBS and afterwards merged with adherent fraction, centrifuged again at 650 g for 5 min at RT and then resuspended in fresh cell culture medium. The CTC-MCC-41 medium was changed every week. HT29 cells were cultured in Dulbecco’s Modified Eagle Medium (DMEM) (#41965-039, Thermo Fisher Scientific Inc., Waltham, MA, USA) supplied with 10% FCS and 1% penicillin/streptomycin mix. Cell culture media were changed twice a week. HEK293T cells were cultivated in DMEM supplied with 10% FCS, 1% penicillin/streptomycin mix, 2 mM L-glutamine, 1 mM sodium-pyruvate (#S8636, Sigma Aldrich, St. Louis, MO, USA) and 20 mM HEPES (#15630, Thermo Fisher Scientific Inc., Waltham, MA, USA). Cultivation medium was refreshed two to three times a week. For transduced cell lines selection antibiotics in an appropriate concentration were maintained in standard cell media to prevent growth of not transduced cells. All cells were grown at 37 °C, 5% CO_2_. All used cell lines were regularly tested for mycoplasma contamination. 

### 4.2. Stable AKT Isoform Specific Knockdown

HEK293T cells were transfected with specific shRNAs purchased from Sigma-Aldrich (St. Louis, MO, USA) targeting either AKT isoforms (pLKO.1-AKT1-puromycin, pLKO.1-AKT2-puromycin, pLKO.1-AKT3-puromycin (2 µg)) or scrambled control (pLKO.1-shRNA-puromycin (2 µg)). Packaging plasmids for third generation lentiviral transfection (pCMV-VSV-G (2 µg), pMDLG/PRE (10 µg), pRSV-REV (5 µg)) were used. The transfection rate was enhanced using the Lipofectamine 3000 transfection reagent according to the manufacturer’s instructions (#L3000-008, Thermo Fisher Scientific Inc., Waltham, MA, USA). The target cell line was seeded in a density of 2 × 10^5^ cells into six well culture plates the day before. Every 24 h the viral particles were collected, filtered through a 0.22 µm filter and 2 mL of the supernatant was added to the target cell line CTC-MCC-41. This step was subsequently repeated three times. Transduction was enhanced using 16 µg hexadimethrine bromide (#H9268, Sigma Aldrich, St. Louis, MO, USA). On the fourth day of transduction the cells were selected by adding 1.5 µg/mL Puromycin to the cell culture media for at least two weeks before cells were used for experiments. Successful isoform specific knockdown of the AKT isoforms was confirmed in western blot analysis prior to further experiments.

### 4.3. Proliferation Assay and Therapeutic Drug Sensitivity Testing

The CTC-MCC-41 line was treated as described above and seeded in technical triplicates in a density of 3000 cells per well into a 96 well flat bottom cell culture plate (Greiner Bio-One, Kremsmünster, Austria). The cells were allowed to settle down at 37 °C, 5% CO_2_ overnight. The next day, the plate was placed into the IncuCyte^®^ Zoom Live Cell Analysis System (Essen Bioscience, Ann Arbor, MI, USA) and cell confluence was measured every 2 hours. Analysis of data was performed based on cell confluence using the provided manufacturer’s IncuCyte^®^ Zoom Software. Statistical analysis of acquired data was carried out using GraphPad Prism 8 (GraphPad Software Inc., San Diego, CA, USA). For the therapeutic drug sensitivity testing the CTC-MCC-41 line was prepared as written above for the proliferation assay. The next day, freshly prepared serial dilutions of either AKT inhibitor MK2206 (#S1078, Selleck Chemicals, Houston, TX, USA), mTOR inhibitor RAD001 (#S1120, Selleck Chemicals, Houston, TX, USA), the combination of both drugs or vehicle dimethyl sulfoxide (DMSO) (#D5879, Sigma-Aldrich, St. Louis, MO, USA) were added to the cells. Measurement and calculation using the IncuCyte^®^ Zoom system was carried out as described in the proliferation section above. 

### 4.4. Apoptosis Assay

5 × 10^5^ cells of either CTC-MCC-41 scrambled (SCR) control, CTC-MCC-41 AKT1 knockdown, CTC-MCC-41 AKT2 knockdown or CTC-MCC-41 stably expressing the AKT3 knockdown vector were plated in 6 well culture plates in biological quadruplicates two days before analysis. 2 µM of Staurosporin (#S5921, Sigma-Aldrich, St. Louis, MO, USA) was added as a positive control 48 h prior to measurement. Unstained CTC-MCC-41 scrambled was used as negative control. The cells were then transferred to the incubator at 37 °C, 5% CO_2_ for 48 h. After 48 h, the suspension cells were removed, adherent cells carefully washed with PBS and detached with 0.05% trypsin-EDTA (#25300-054, Thermo Fisher Scientific Inc., Waltham, MA, USA). Afterwards the adherent and suspension cells were centrifuged at 1900 rpm for 5 min at room temperature. The cells were resuspended in 100 µL 1× binding buffer supplied within the FITC Annexin V Apoptosis Detection Kit (#556547, BD Biosciences, Franklin Lakes, NJ, USA) and transferred to Falcon^®^ round-bottom polystyrene tubes (Corning Inc., New York, NY, USA). Cells were stained with propidium iodide, FITC Annexin V or the combination of both dyes according to manufacturer’s instructions. The FACS measurement was performed on BD LSRFortessa (BD Biosciences, Franklin Lakes, NJ, USA) and laser settings precisely improved for CTC-MCC-41 cells using the unstained control. Data was analyzed with FlowJo version 10.6.1 (BD Biosciences, Franklin Lakes, NJ, USA). Statistical analysis of the data was performed as described in the statistical analysis section.

### 4.5. Western Blot Analysis and Densitometric Quantification

Cell lysates were prepared in NP-40 lysis buffer containing 50 mM HEPES (#H3375, Sigma-Aldrich, St. Louis, MO, USA) at pH 7.5, 150 mM NaCl (#0278, J.T. Baker, Thermo Fisher Scientific Inc., Waltham, MA, USA), 1% NP-40 (#98379, Sigma-Aldrich, St. Louis, MO, USA), 2% Aprotinin (#A162.2, Carl Roth GmbH + Co. KG, Karlsruhe, Germany), 2 mM EDTA (#E5134, Sigma-Aldrich, St. Louis, MO, USA), 50 mM NaF (#S7920, Sigma-Aldrich, St. Louis, MO, USA), 10 mM NaPPi (#S6422, Sigma-Aldrich, St. Louis, MO, USA), 10% Glycine (#3908, Carl Roth GmbH + Co. KG, Karlsruhe, Germany), 1 mM Sodium orthovanadate (#S6508, Sigma-Aldrich, St. Louis, MO, USA) and 1 mM PMSF (#10837091001, Sigma-Aldrich, St. Louis, MO, USA). Protein determination was conducted using a lowry protein assay (#5000112, Bio-Rad Laboratories Inc., Hercules, CA, USA). Proteins were separated in SDS page gel electrophoresis and transferred to a nitrocellulose membrane. Afterwards the membranes were incubated with specific primary antibodies against mTOR, pmTOR (S2448), pAKT (S473), AKT (pan), AKT1, AKT2, AKT3, pERK1/2 (T202/Y204), ERK1/2, pS6 (S240/S244), S6 (all Cell Signaling Technology Inc., Danvers, MA, USA) or HSC70 (Santa Cruz Biotechnology Inc., Dallas, TX, USA). Species specific horseradish peroxidase linked secondary antibodies against anti-rabbit IgG or anti-mouse IgG were purchased from Santa Cruz Biotechnology Inc (Dallas, TX, USA). Equal protein loading was ensured by 0.2% Ponceau S staining (#33427, Serva Electrophoresis GmbH, Heidelberg, Germany) and housekeeping protein HSC70. For more information on antibodies used in the experiments please see the supplementary material (Supplementary Table S1). Western blot images were acquired using the ImageQuant LAS 4000 system (GE Healthcare Bio-Sciences, Pittsburgh, PA, USA). Densitometric quantification of the western blots (*n* = 3) was done with the AIDA Image Analysis software (Elysia-raytest GmbH, Straubenhardt, Germany). Statistical analysis of the densitometric quantification was performed in GraphPad Prism 8 (GraphPad Software Inc., San Diego, CA, USA) as described in the statistical analysis section below. 

### 4.6. Calculation of Combination Indices

Combination indices (CI) for drug combinations were calculated according to the Chou and Talalay method using CompuSyn software (ComboSyn Inc., Paramus, NJ, USA). CI values were encoded as recommended by Chou and Talalay [43]. Very strong synergism CI < 0.1; strong synergism CI 0.1–0.3; synergism CI 0.3–0.7; moderate synergism 0.7–0.85; slight synergism CI 0.85–0.90; nearly additive CI 0.9–1.10; CI values > 1.1 were considered as antagonism.

### 4.7. Statistical Analysis

All statistical analyses were performed with implemented functions in GraphPad Prism 8.4.1 (GraphPad Software Inc., San Diego, CA, USA). A *p* value < 0.05 was considered as statistically significant. *p* values were encoded into asterisks as following: ns *p* > 0.05; * *p* ≤ 0.05; ** *p* ≤ 0.01; *** *p* ≤ 0.001; **** *p* ≤ 0.0001. 

## 5. Conclusions

The PI3K/AKT/mTOR axis plays a crucial role in colorectal carcinoma (CRC). Here, we demonstrate a pivot role of this signaling pathway in the CTC-MCC-41 line, which is derived from a patient with colorectal cancer. CTC-MCC-41 is highly sensitive for dual inhibition of AKT/mTOR using MK2206 and RAD001 in vitro and exhibits strong synergistic effects within the nanomolar range in therapeutic drug screening assays. Strong activation of the PI3K/AKT/mTOR pathway indicated by phosphorylation of AKT and downstream substrates could be observed. Furthermore, AKT isoform specific knockdown of AKT1 and AKT2 significantly diminished proliferation of the cell line in comparison to control, suggesting a crucial role of the PI3K/AKT/mTOR pathway in CTC-MCC-41. Therefore, these data may indicate that the inhibition of the PI3K/AKT/mTOR pathway in CTCs may be a valuable target to block metastatic progression in CRC patients that should be confirmed in further xenograft mouse models in vivo. 

## Supplementary Materials

The following are available online at https://www.mdpi.com/2073-4409/9/9/2129/s1, Supplementary Figure S1: Effect of AKT isoform specific knockdowns on apoptosis of CTC-MCC-41 cells, Supplementary Table S1: Primary and secondary antibodies used for Western blot analysis.
