# Supplementary material for: High Sensitivity of Circulating Tumor Cells Derived from a Colorectal Cancer Patient for Dual Inhibition with AKT and mTOR Inhibitors
Source: Cells. 2020 Sep 20;9(9):2129. doi: 10.3390/cells9092129 (PMC7566012; doi:10.3390/cells9092129)
Supplement: Supplementary file 1 [file cells-09-02129-s001.pdf]

Supplementary Material:

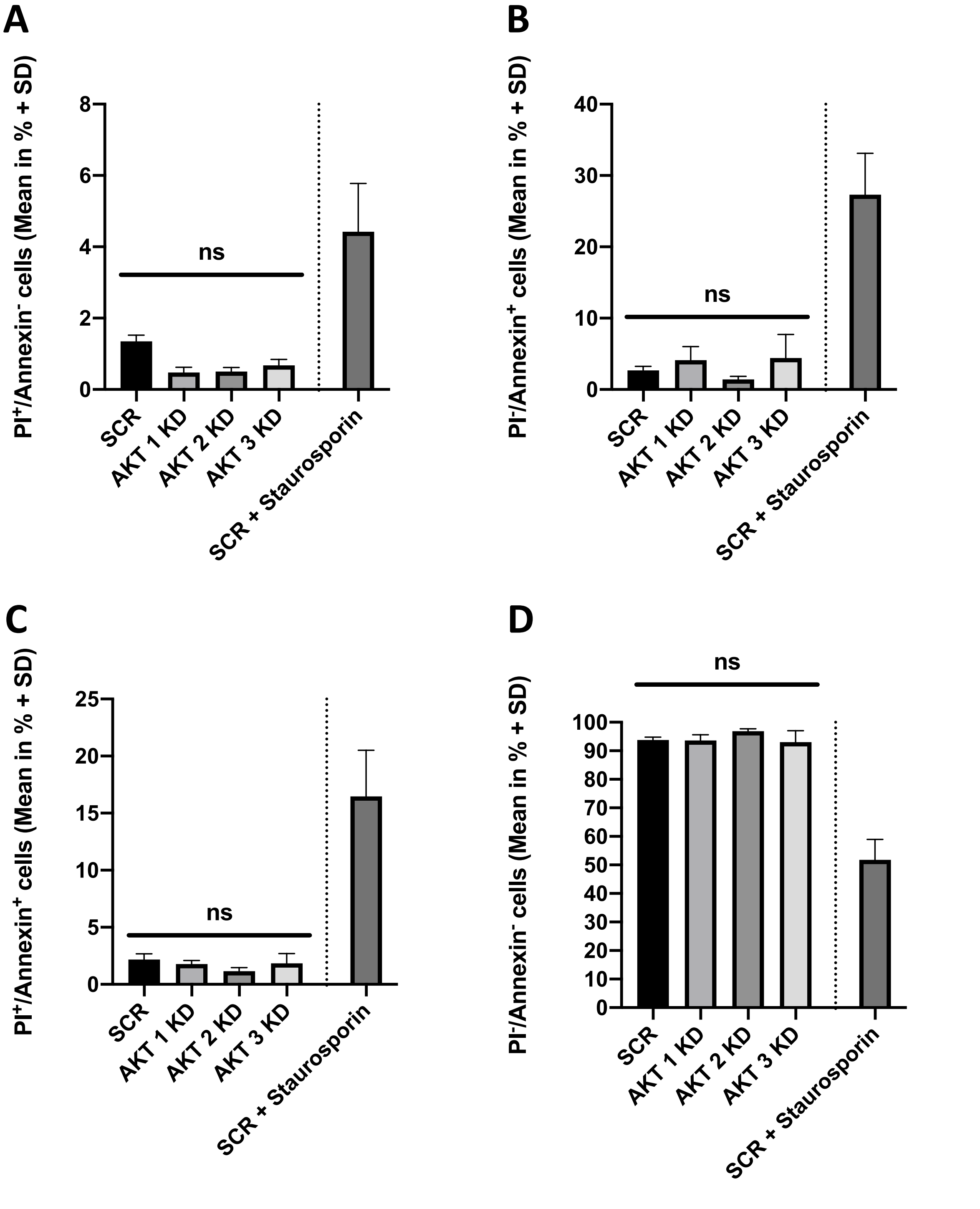

Supplementary Figure S1. Effect of AKT isoform specific knockdowns on apoptosis of CTC-MCC-41 cells. Stable isoform specific knockdowns of AKT and SCR control were subjected to FACS analysis. 2 μM Staurosporin was used to induce apoptosis as a positive control in CTC-MCC-41 SCR cells. Cells were maintained for 48 hours and then stained with either propidium iodid (PI) (A), Annexin V (FITC) (B) or the combination of both agents (C). Unstained control can be found in (D). The cells were subjected to FACS analysis in biological quadruplicates. The cells were gated for single cells prior to analysis. p values were calculated using one-way ANOVA with Tukey's multiple comparisons test (ns $p > 0.05$). The mean values ($n = 4$) with standard deviation are shown.

Supplementary Table S1. Primary and secondary antibodies used for western blot analysis.

Target	Manufacturer	Clone / Reference number	Host species	Dilution
AKT1	Cell Signaling Technology	C73H10, #2938	rabbit	1:1000
AKT2	Cell Signaling Technology	5B5, #2964	rabbit	1:1000
AKT3	Cell Signaling Technology	62A8, #3788	rabbit	1:1000
Anti-mouse IgG, HRP-linked	Cell Signaling Technology	#7076	horse	1:5000
Anti-rabbit IgG, HRP-linked	Cell Signaling Technology	#7074	goat	1:5000
HSC70	Santa Cruz Biotechnology	B-6, #sc-7298	mouse	1:1000
p44/42 MAPK (Erk1/2)	Cell Signaling Technology	#9102	rabbit	1:1000
mTOR	Cell Signaling Technology	7C10, #2983	rabbit	1:1000
panAKT	Cell Signaling Technology	11E7, #4685	rabbit	1:1000
pAKT (S473)	Cell Signaling Technology	D9E, #4060	rabbit	1:1000
pmTOR (S2448)	Cell Signaling Technology	#2971	rabbit	1:1000
pp44/42 MAPK (Erk1/2) (T202/Y204)	Cell Signaling Technology	#9101	rabbit	1:1000
pS6 ribosomal protein (S240/S244)	Cell Signaling Technology	D68F8, #5364	rabbit	1:1000
S6 ribosomal protein	Cell Signaling Technology	5G10, #2217	rabbit	1:1000
